# Three-dimensional visualisation of human ovarian follicles using a whole-mount immunolabelling and optical tissue clearing method

**DOI:** 10.1530/RAF-25-0096

**Published:** 2025-10-31

**Authors:** A S Harrison, B D Bjarkadottir, N Kuscu, J Davies, S Lane, S A Williams

**Affiliations:** ^1^ Nuffield Department of Women’s & Reproductive Health, University of Oxford, Women’s Centre, John Radcliffe Hospital, Oxford, United Kingdom; ^2^ Oxford Cell & Tissue Biobank, Oxford University Hospitals NHS Foundation Trust, Oxford, United Kingdom

**Keywords:** human, ovary, follicle, optical tissue clearing, three-dimensional imaging, immunolabelling

## Abstract

**Abstract:**

To more accurately classify human ovarian follicles by developing a novel methodology with three-dimensional visualisation using whole-mount immunolabelling and optical tissue clearing techniques. A whole-mount immunolabelling protocol combined with optical tissue clearing was applied to human ovarian tissue for three-dimensional follicle analysis. Key markers, including DAPI for nuclear staining, DDX4 for oocyte-specific labelling, and LAMININ for basal membranes, were used to visualise follicular structures. The tissues underwent a clearing process to enable confocal z-stack imaging to obtain detailed three-dimensional data. Ovarian cortical tissue from three paediatric patients (aged 9–12 years) who had not undergone chemotherapy treatment was collected. The tissue was dissected into small fragments and stained to identify viable follicles. Follicle and oocyte size, as well as granulosa cell size and type, were assessed across multiple dimensions (XY, XZ, YZ planes). Follicles were classified as primordial or transitional based on granulosa cell characteristics. Three-dimensional analysis across XY, XZ, and YZ planes revealed that 58% of follicles, initially classified as primordial using conventional two-dimensional methods, were misidentified as transitional due to the presence of cuboidal granulosa cells that were only detectable in multi-plane analysis. Moreover, three-dimensional analyses revealed significant differences in the number and size of cuboidal granulosa cells detected on transitional follicles. This study presents a novel whole-mount immunolabelling and optical tissue clearing methodology for accurate three-dimensional visualisation and classification of human ovarian follicles. This technique improves the accuracy of follicle staging and provides a valuable tool for future research into ovarian function, reproductive health, and conditions impacting follicle development.

**Lay Summary:**

In this study, we explored a new method for examining ovarian follicles – structures that contain the immature eggs – within human ovarian tissue. Traditional methods, which rely on two-dimensional imaging, can lead to mistakes in classifying the development of these follicles due to their spherical shape. To address this, we developed techniques to visualise the ovarian tissue in three dimensions. Using ovarian tissue from three patients, the tissue was processed using techniques called whole-mount immunolabelling (to detect structures) followed by optical clearing (to visualise the structures) and then analysed using microscopy. This enabled us to create detailed 3D images that showed the structures of the immature eggs and follicles across different planes of view. We discovered that conventional 2D assessments misclassified nearly 60% of the follicles, as certain developmental characteristics were only visible in the 3D images. This innovative approach marks the first time such detailed 3D imaging has been applied to human ovarian follicles. It not only enhances the accuracy of classifying follicle stages but also holds potential for improving fertility preservation techniques in the future. Overall, the study highlights the benefits of using modern imaging methods to gain better insights into ovarian health and development.

## Introduction

**Figure 1:**
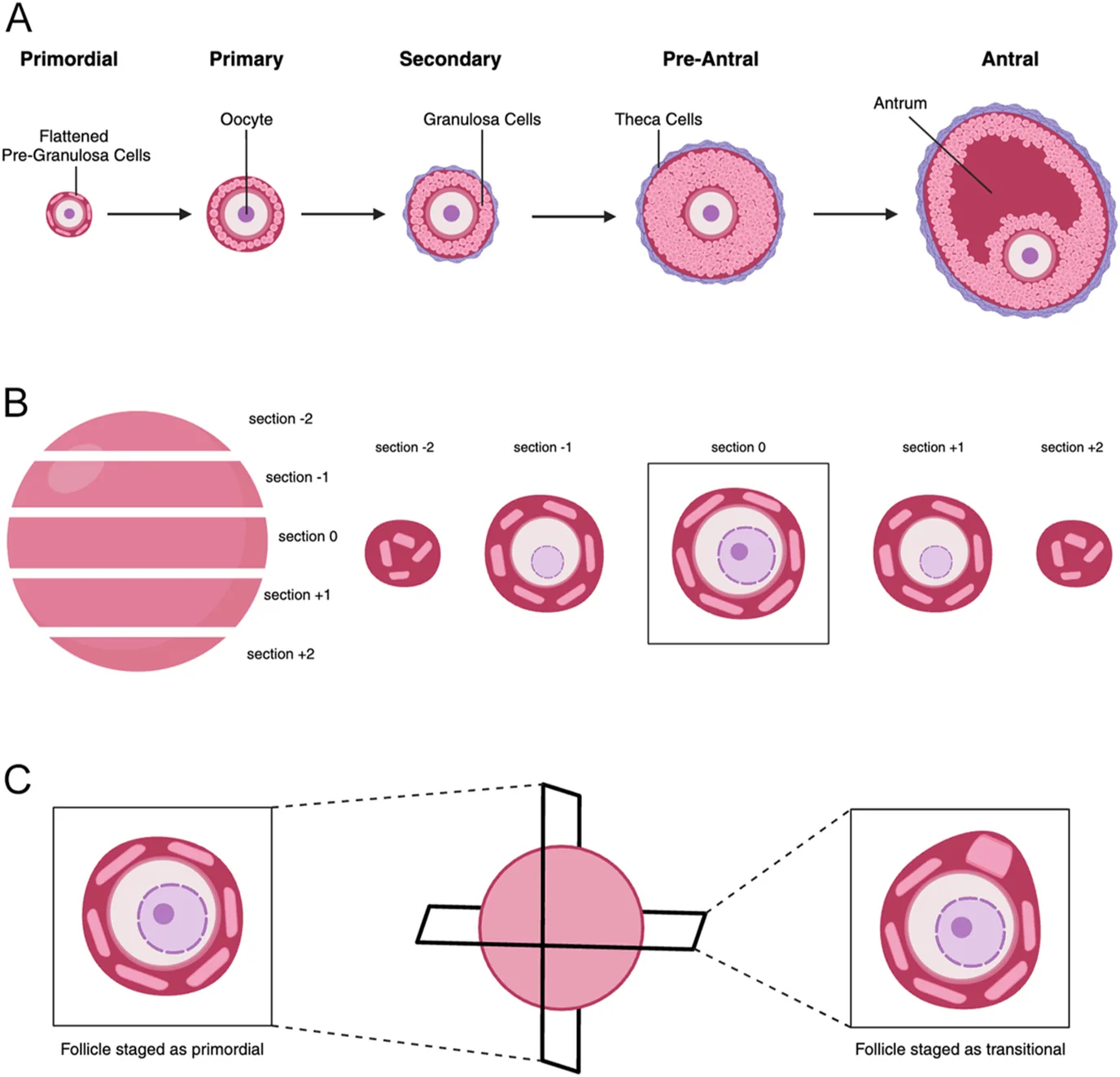
Overview of follicle development and standard histological analysis limitations. (A) The primordial follicle is the earliest stage of development, consisting of a single oocyte surrounded by a single layer of flattened pre-granulosa cells. Following follicle activation, the flattened pre-granulosa cells (fpGCs) begin to divide and become cuboidal granulosa cells (GCs). Once a full single layer of GCs is present, it is then considered a primary follicle. At this stage, the glycoprotein layer, called the zona pellucida, begins to form around the oocyte. A follicle is considered secondary when two full layers of GCs surround the oocyte; the theca cells form a layer around the follicular basal lamina. When three or more full layers of GCs surround the oocyte, the follicle is pre-antral. The antrum, a fluid-filled cavity, is a defining feature of the gonadotropin-dependent antral follicle. The follicle will continue to grow (∼20 mm in humans) before ovulation. (B) Diagram of serial histological sections through a primordial follicle, shown as both a three-dimensional sphere and corresponding individual tissue sections. Only the largest follicle cross-section (represented by ‘section 0’) containing the oocyte nucleus is analysed. The sections before the largest follicle cross-section (represented by ‘section −2’ and ‘section −1’) and following the largest follicle cross-section (represented by ‘section +1’ and ‘section +2’) are not analysed. Thus, information is lost as the fpGCs contained in the neighbouring sections are disregarded. (C) As follicles cannot be visualised within ovarian tissue, the orientation of the follicle and the angle when sectioning cannot be controlled for. Thus, transitional follicles could be misclassified as primordial. For example, the largest resulting follicle cross-section when cut along the y-axis is staged as primordial (left). The largest cross-section of the same follicle when cut along the x-axis is staged as a transitional follicle due to the presence of a single cuboidal GC (right). Images are not to scale. Created with BioRender.com.

The ovary is a heterogeneous organ, consisting of an outer cortex and an inner medulla, with follicles, blood vessels, and stroma. Ovarian follicles are the reproductive unit of the ovary, containing a single egg, herein referred to as the oocyte, surrounded by specialised somatic cells, including theca and granulosa cells (GCs) (Fig. 1A). Bidirectional communication between the oocyte and its neighbouring cells is vital for follicle development. Analysis of follicle development is reliant upon the intact morphology, location, and quantity of GCs surrounding the oocyte. Classification is important for understanding the stages of follicle maturation, which is crucial for reproductive health, managing fertility treatments, and conducting studies on ovarian function.

Histology and immunohistochemistry (IHC) are considered gold-standard methods to analyse early follicle development because they support the visualisation of structures within tissue. Methods that require cell digests or tissue lysates, such as Western blotting, can destroy cellular structures, preventing ovarian follicle visualisation. IHC enables the specific identification and localisation of proteins, providing valuable insights into the distribution and expression patterns of target molecules. Histology and IHC routinely involve the preparation of thin sections of tissue (typically 5 μm thick), which are then stained or immunolabelled and visualised under a brightfield microscope. A significant limitation is that a 5 μm section cannot encapsulate the three-dimensional follicle structure, which can range in size from ∼40 μm for primordial follicles to over 18 mm for a pre-ovulatory follicle in humans (Griffin *et al.* 2006).

Rigorous distinction between follicles is important for accurate analysis. In standard practice, the largest cross-section of each follicle, identified by the presence of the oocyte nucleus, is used for assessment. Although time-efficient, the approach of assessing one section per follicle can lead to errors. First, only follicular cells within the chosen section are included for follicle analysis, while cells in neighbouring sections of the same follicle are excluded (Fig. 1B). Therefore, follicles could only be assessed as ‘atretic’ or ‘growing’ based on the presence or absence of pyknotic or mitotic GCs, due to the limited information provided by one section (Uslu *et al.* 2017). Second, follicles are classified into stages for subgroup analysis based on morphological data within the specific cross-section (Fig. 1C). There is no rationale to orient the tissue for optimal cross-section representation because follicles cannot be seen externally within intact tissue during the embedding process. There is potential for variable characteristics to be identified depending on the angle and orientation of the cross-section. Theoretically, a follicle classified as primordial due to the surrounding flattened pre-granulosa cells (fpGCs) in the analysed cross-section may contain concealed cuboidal cells at the opposite poles. Thus, the follicle would be more appropriately staged as transitional.

Accurate classification of early-stage follicles is essential for understanding ovarian reserve regulation. The transitional follicle stage marks the earliest morphological and molecular signs of activation from the dormant primordial stage (Maciel *et al.* 2004, McLaughlin & McIver 2009, Zhang *et al.* 2018, Rooda *et al.* 2024, Kubo *et al.* 2025). Follicular activation is tightly controlled, underpinning both the reproductive lifespan and fertility potential (Yang *et al.* 2020, Telfer & Andersen 2021). Improved methods for classifying early-stage follicles allow for more precise assessment of follicle dynamics, ovarian health, and the impact of clinical interventions such as chemotherapy (Titus *et al.* 2021, Erden & Oktay 2025).

Recent advancements in imaging and microscopy may assist in offering a solution for thorough follicle classification. Confocal microscopy enables high-resolution imaging at different focal planes in the z-plane through ‘optical sectioning’ without physically slicing the sample (Conchello & Lichtman 2005). Alternative non-invasive methods to visualise human ovarian follicles within tissue pieces include full-field optical coherence tomography (Peters *et al.* 2016) and laser-scanning confocal microscopy with rhodamine 123 staining of active mitochondria in follicles (Soleimani *et al.* 2006).

However, factors such as light scattering limit the imaging of whole tissue samples. Light scattering is characterised by light repeatedly reflecting of cellular structures, creating a milky appearance. This is due to the mismatch in refractive index, a measure describing the speed of light through a material, between the sample and the immersion medium, causing light to bend as it passes through the material (Richardson & Lichtman 2015). For example, the mismatch of refractive index between air (1.00) and water (1.33) can be observed as light is refracted when moving through a glass of water, causing an object, such as a straw, to appear bent.

Optical clearing techniques have been developed to allow imaging of tissue at greater z-depths by mitigating factors such as light scattering (Richardson & Lichtman 2015). Solvent-based tissue clearing involves tissue dehydration to remove water and some lipids, followed by immersion in a clearing agent such as benzyl benzoate, which alters the sample’s refractive index to match that of the immersion medium (Richardson & Lichtman 2015). Tissue-clearing methods have been implemented on mammalian reproductive organs (Soygur & Laird 2021, Folts *et al.* 2023), including the placenta (human and non-human primate; Merz *et al.* 2018, Sargent *et al.* 2019) and ovary (mouse; Faire *et al.* 2015, Feng *et al.* 2017, Kagami *et al.* 2018, Rinaldi *et al.* 2018, McKey *et al.* 2020, Tong *et al.* 2020, Boateng *et al.* 2021, Soygur *et al.* 2023) (fish; Lesage *et al.* 2020) (human; Biswas *et al.* 2021, Barbato *et al.* 2023). Tissue clearing has been implemented on developing embryos (human and mouse; Belle *et al.* 2017, Bunce *et al.* 2020), as well as external genitalia (foetal human; Isaacson *et al.* 2020) and uterus (adult mouse and human; Arora *et al.* 2016). At the time of reporting, there have been no reports of optical tissue clearing of human ovarian tissue for classification of follicles.

In this study, we have developed a methodology that enables the three-dimensional visualisation and classification of ovarian follicles in human ovarian tissue for the first time. Follicles, as early as the primordial stage, can now be accurately analysed via the whole-mount immunolabelling and tissue clearing method.

## Materials and methods

### Ethics

The use of human ovarian tissue was approved by the Health Research Authority South Central – Oxford B Research Ethics Committee under REC licence (REC reference 19/NW/0526). Tissue was collected from the Oxford Cell and Tissue Biobank located at the John Radcliffe Hospital, Oxford, UK. All patients, or their legal guardians, provided informed consent before ovarian tissue collection.

### Patient samples

Fresh ovarian cortical tissue was collected from three patients (A, B, and C) who were 9, 11, and 12 years of age at the time of donation, respectively. Patients were chemotherapy-naïve at the time of donation. Patient A received a diagnosis of osteosarcoma, patient B had a diagnosis of sickle cell disease, and patient C had received a diagnosis of localised synovial sarcoma. All three paediatric patients contributed ovarian cortical tissue for method development. Fragments from patient A and patient C were used for the purposes of protocol development and optimisation; follicles analysed and presented here were derived from patient B.

### Ovarian tissue dissection

Human ovarian cortical strips were transferred to dissection medium, consisting of Leibovitz’s L-15 (L5520, Sigma-Aldrich, UK) medium supplemented with 3 mg/mL human serum albumin (HSA; AI653, Sigma-Aldrich), 100 U/mL penicillin, 100 μg/mL streptomycin (Penicillin-Streptomycin; P0781, Sigma-Aldrich), 2 mM L-glutamine (25030024, Life Technologies, UK), and 2 mM sodium pyruvate (S8636, Sigma-Aldrich). The tissue was kept at 4°C until use (within 1 h) and processed into fragments. Cortical tissue strips in dissection medium were cut using scalpel blades (No. 15, 233-5366, and No. 24, 233-5529, VWR International, UK) into fragments ∼0.25 mm thick (approximately 0.5 × 0.5 × 0.25 mm) under a stereomicroscope (M125, Leica Microsystems, UK).

Processed fragments were stained with 50 μg/mL neutral red (NR; N2889, Sigma-Aldrich) in minimal essential medium alpha (αMEM; 22561021, Thermo Fisher) containing the same supplements as the dissection medium to identify pieces containing viable follicles, as previously described (Walker *et al.* 2021). Stained fragments were incubated in a humidified incubator (5% CO_2_) for 2 h at 37°C. Following incubation, fragments were visualised under a dissection microscope. Visible, red-stained follicles (NR-positive) were preferentially selected for culture. Tissue pieces were washed briefly in dissection medium before culture.

### Ovarian tissue culture

All tissue fragments included in this study were cultured before fixation, clearing, and imaging. Ovarian tissue pieces were cultured individually in Corning Costar 24-well culture plates (3526, Scientific Laboratory Supplies, UK) in 300 μL culture medium, consisting of αMEM supplemented with 1 mg/mL HSA, 100 U/mL penicillin, 100 μg/mL streptomycin, 2 mM L-glutamine, 10 μg/mL insulin, 5.5 μg/mL transferrin, 5 ng/mL selenium (I3145, Sigma-Aldrich), 50 μg/mL ascorbic acid (10012011, Thermo Fisher, UK), and 12.5 IU/L recombinant human follicle-stimulating hormone (Gonal-f; Z1540, Merck Serono, UK). Sterile water supplemented with 100 U/mL penicillin and 100 μg/mL streptomycin was added to the inter-well space to prevent medium evaporation. All culture medium was equilibrated for at least 1 h in an incubator before use. Ovarian tissue was cultured for up to 48 h; culture was carried out before analysis to ensure that our method was compatible with cultured tissues.

Following culture, samples were fixed in 1% form-acetic, a fixative consisting of 10% neutral buffered formalin with 1% acetic acid, which reduces the formation of fixation-induced artefacts; form-acetic has been previously described (Adeniran *et al.* 2021). Tissue was fixed at room temperature (RT) on a rocker for 24 h, after which tissue was transferred to 70% ethanol and stored at 4°C until processing.

### Whole-mount immunolabelling protocol

The protocol was developed to allow visualisation of human ovarian tissue fragments using a commercially available Tissue Clearing Kit (ab243298, Abcam, UK).

### Washing and permeabilisation

Fixed tissue pieces were washed twice in phosphate-buffered saline (PBS; 20 mM phosphate, 150 mM NaCl, pH 7.4) and incubated for 15 min each. All incubations were completed at 4°C with gentle agitation unless otherwise indicated. The tissue was dehydrated and incubated in an increasing series of methanol solutions: 50% methanol/PBS, 80% methanol, and 100% methanol. Tissue was permeabilised and incubated in 20% dimethyl sulfoxide (DMSO; D8418, Sigma) solution with methanol. Following permeabilisation, samples were rehydrated and incubated in 80% methanol, 50% methanol/PBS, then 100% PBS solution. Finally, samples were then incubated in a penetration buffer (PBS with 0.2% Triton X-100, 0.3 M glycine, and 20% DMSO) overnight at RT with gentle agitation.

### Whole-mount immunolabelling

The next day, tissues were transferred to a 400 μL blocking solution (PBS with 0.2% Triton X-100, 6% donkey serum, and 10% DMSO) and incubated for 24 h. Tissues were labelled with rabbit anti-LAMININ polyclonal antibody (1.75 μg/mL, ab11575, Abcam) as a marker for the basal lamina and rabbit anti-DEAD-Box Helicase 4 (DDX4) polyclonal antibody (10 μg/mL, ab13840, Abcam) as an oocyte-specific marker. Both were diluted in antibody buffer (PBS with 0.2% Tween, heparin, 3% donkey serum, and 5% DMSO; total volume 400 μL) for 7 days at 4°C (the incubation time required was determined experimentally; data not shown). Following primary antibody incubation, tissues were washed five times for 20 min in washing buffer (PBS with 0.2% Tween and heparin), after which they were transferred to 400 μL of secondary antibody solution (Alexa Fluor 488 donkey anti-rabbit IgG; 1:100, 20 μg/mL, A21205, Thermo Fisher Scientific) to detect LAMININ and DDX4. A negative control was prepared using rabbit IgG (10 μg/mL, 3900S, Cell Signaling Technology, USA) and mouse IgG1 (14.2 μg/mL, 401401, BioLegend, USA). Nuclei were counterstained using 5 μg/mL 4′,6-diamidino-2-phenylindole (DAPI; D9542, Sigma-Aldrich) in antibody buffer. Tissues were incubated for 7 days at 4°C and protected from light.

### Tissue clearing

All following steps were performed while protecting samples from light. Tissues were washed ten times in washing buffer for 10 min at RT with gentle agitation. Tissues were then dehydrated by washing in 50% methanol/PBS, 80% methanol, and three washes of 100% methanol for 15 min each at RT with gentle agitation. Tissues were removed from methanol and carefully dried (using lens paper and allowing the methanol to evaporate).

**Figure 2:**
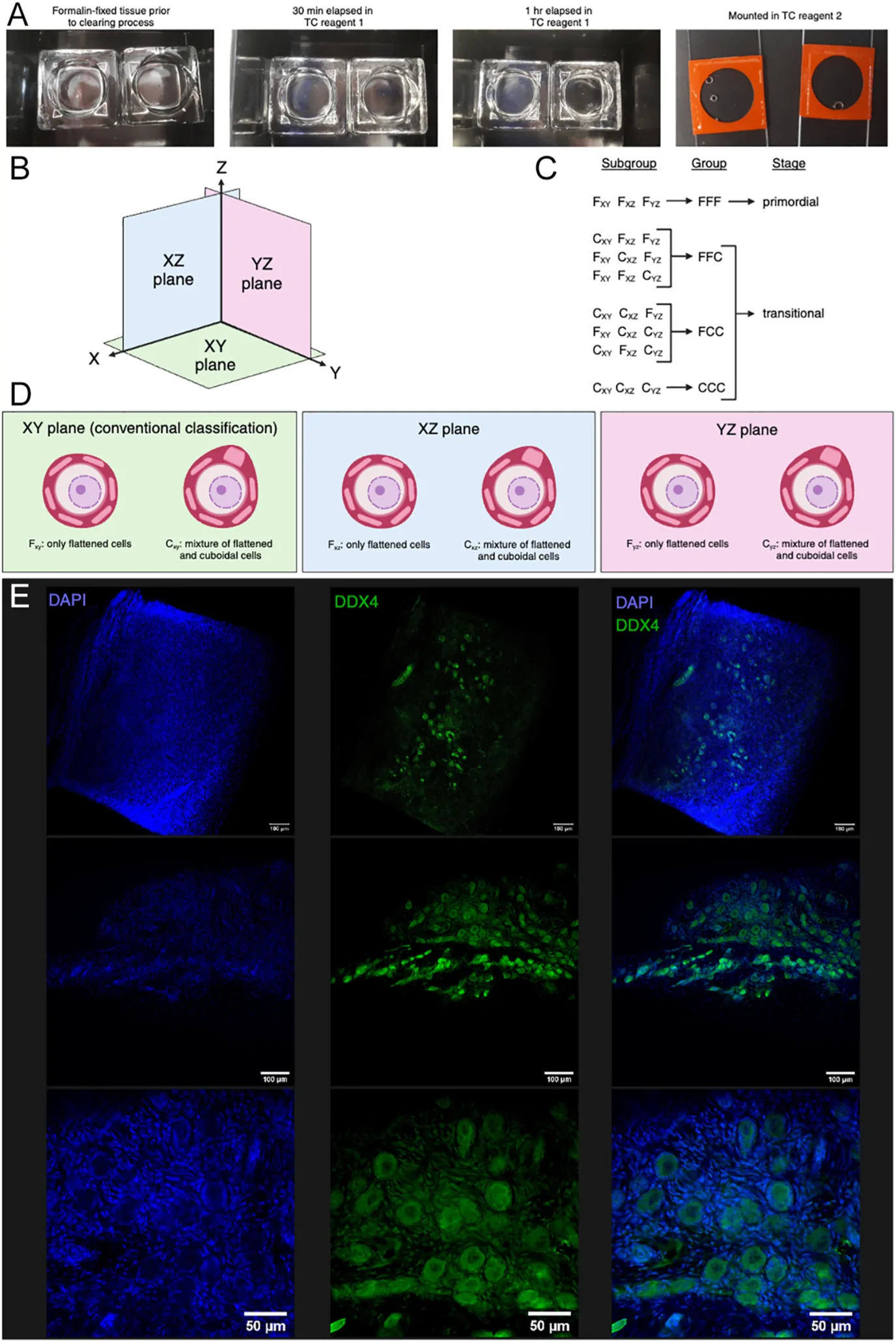
Establishment of a novel method to optically clear, whole-mount immunolabel, and three-dimensionally classify ovarian follicles in human tissue. (A) A fragment of human ovarian tissue stained with 5 μg/mL DAPI nuclear counterstain (L-R) in an embryo dish before optical clearing using Visikol® HISTO™ technology, after 30 min in tissue clearing (TC) reagent 1, after 1 h in TC reagent 1, and the tissue near-invisible after mounting on glass coverslips in TC reagent 2. Arrowheads denote the fragment. (B) Diagram of the three coordinate planes: XY (green), YZ (pink), and XZ (blue). (C) Each follicle could be classified into one of eight subgroups (left column) based on morphological classification across each of the three planes. Follicles were grouped based on having flattened pre-granulosa cells across all three planes (FFF), or as having cuboidal granulosa cells at one (FFC), two (FCC), or all three planes (CCC). Follicles in the FFF group were classified as primordial, while follicles in the FFC, FCC, or CCC groups were considered transitional stage. (D) Each follicle was assessed across three planes and categorised based on morphology as having only flattened pre-granulosa cells (F) or a mixture of flattened pre-granulosa cells and cuboidal granulosa cells (C). Subscript indicates the relevant plane. Histological analysis, conventional classification, is completed on the XY plane. (E) Whole-mount immunolabelling of DEAD-Box Helicase 4 (DDX4) (1:100) antibody as a follicular marker (oocyte) in optically cleared human ovarian tissue (<250 μm thickness), as seen in green. Samples were counterstained with DAPI (blue). A single human ovarian tissue sample is shown with DAPI, DDX4, and merged at three distances. Optical sectioning (10 μm intervals) imaged with a 10× objective. Created with BioRender.com.

After dehydration in methanol, tissues were transferred to a glass embryo dish containing 1 mL Tissue Clearing Reagent 1 (refractive index of 1.50; mixture of benzyl alcohol and 2,2,2-trichloroethanol) and incubated for 1 h at RT with gentle agitation (Fig. 2A). Tissues were mounted in a prepared silicone isolator (approximately 13 mm diameter and 0.25 mm deep) on a glass coverslip (22 × 50 mm, #1.5) filled with Tissue Clearing Reagent 2 (refractive index 1.50; mixture of diphenyl ether, 2,2,2-trichloroethanol, and benzyl alcohol). Tissues were placed in the centre of the well and sealed with a square coverslip (22 × 22 mm, #1.5). Tissues were stored at 4°C away from light until imaged. Tissue clearing could be reversed by incubating cleared samples in 100% ethanol at RT until sample opacity was restored.

### Imaging

Images were collected using a Zeiss 780 upright confocal microscope. Images of whole tissue fragments were obtained with a ×10/0.45 numerical aperture objective (working distance 2 mm) or ×25/0.8 numerical aperture oil immersion objective (working distance 570 μm). DAPI was excited using a 405 nm laser and detected at 380–480 nm. Alexa Fluor 488 was excited using a 488 nm laser and detected at 490–555 nm emission. Optimal pinhole size was determined for each channel and kept consistent for all samples. Gain settings for DAPI and Alexa Fluor 488 were adjusted individually for each sample to allow clear visualisation of cell nuclei (DAPI) and LAMININ and oocytes (Alexa Fluor 488), as these markers would not be quantified. Z-compensation with extrapolation was used to adjust for decreasing signal brightness at greater z-depths. Z-stacks were obtained by identifying the top and bottom of the sample. Samples were first imaged from one side and then flipped over and imaged again.

### Image analysis

Follicles were enumerated across cleared fragments meeting inclusion criteria; per-fragment counts were not prospectively recorded for the final analysis set, and results are therefore reported at patient level. Images of cleared tissue samples were analysed using ImageJ Fiji and Imaris (version 9.7.0) of patient B. Measurements across the XY, XZ, or YZ planes were performed in ImageJ Fiji using orthogonal views and the measurement tools to measure the size of follicles, oocytes, and GCs (Fig. 2B, C, D). GCs and fpGCs, with nuclear counterstaining, were visualised as the space between the LAMININ and DDX4 signal to account for variability in cellular expression across different follicular stages. Follicle health and incidence of atresia were not systematically assessed in this study. Oocyte and follicle volume (*V*) was calculated based on the oocyte or follicle radius (*r*) at the *x*, *y*, and *z* planes using the following formula for the volume of an ellipsoid:

$$V=\frac{4}{3}\pi r_{x}r_{y}r_{z.}$$

### Statistical analysis

Data were analysed using GraphPad Prism (version 9.1). Data were tested for normality using the Shapiro–Wilk test. One-way ANOVA with Tukey’s post-hoc testing was used to compare GC, oocyte, and follicle size measurements of primordial and transitional follicles. Statistical significance was defined as *P* < 0.05. Descriptive statistics are presented as counts and percentages.

## Results

### Visualisation of follicles using tissue clearing method

**Figure 3:**
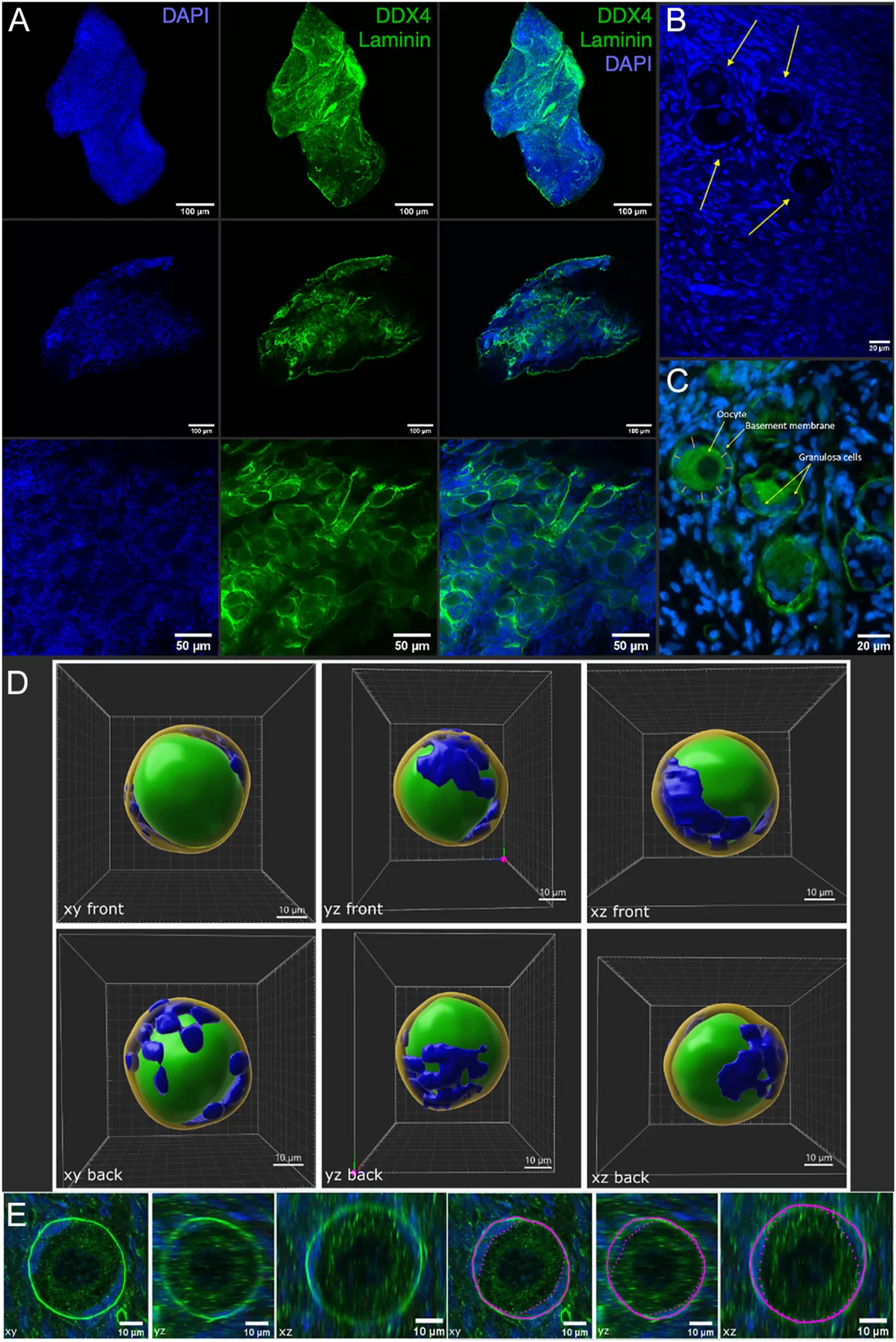
Visualisation and three-dimensional rendering of ovarian follicular structures in optically cleared human tissue. (A, C, E) Human ovarian tissue was labelled with LAMININ and DEAD-Box Helicase 4 (DDX4) to detect the follicle basement membrane and oocyte, respectively, in green. Samples were counterstained with DAPI (blue) and optically cleared. (A) Representative tissue following whole-mount immunolabelling and tissue clearing of a single human ovarian tissue sample labelled with DAPI, DDX4, and merged at three distances. (B) Identification of primordial and transitional follicles (yellow arrows) in a single slice through the centre of the tissue fragment counterstained with DAPI (blue) as a nuclear marker. Oocytes can be seen as black circles with the oocyte nucleus as the dimmer blue circle inside. (C) Follicles, labelled with DDX4 and LAMININ (green) with DAPI counterstain (blue), with structures identified (oocyte, basement membrane, granulosa cells) with yellow arrows. Orange lines represent the distance between the oocyte and the basement membrane wherein the (pre-)granulosa cells are present. (D) Three-dimensional reconstruction, using Imaris, of the same follicle as E. The basal lamina (yellow), oocyte (green), and granulosa cell nuclei (blue) were manually selected, allowing three-dimensional reconstruction. The follicle is shown from different angles (e.g. xy front) as indicated on each panel. (E) Representative follicle labelled with LAMININand DDX4 (green) and DAPI (blue) labelling demonstrating a central slice through the XY plane and orthogonal views through the XZ and YZ planes in three serial images. Granulosa cell (GC) visualisation is highlighted as the area between the basement membrane (magenta, unbroken line) and the oocyte (magenta, dotted line) in repeated images. Imaged with a 10× objective at 10 μm intervals (A) or using a 25× oil immersion objective to obtain z-stacks at 1 μm intervals (B, C, E). Created with BioRender.com.

By implementing the whole-mount immunolabelling and tissue clearing technique with DAPI and DDX4, follicles were successfully visualised in ovarian tissues (Fig. 2E). The resulting optical sectioning in z-stacks allows for the visualisation of follicles throughout the ovarian tissue (Supplementary Video 1 (see section on Supplementary materials given at the end of the article)). Follicular structures, including the oocyte and GCs, can be observed via magnification of z-stack images. The most optimal results for follicle assessment were achieved when utilising a combination of DAPI, DDX4, and LAMININ (a marker for the follicle basal membrane) (Fig. 3A; Supplementary Video 2).

### Three-dimensional staging of follicles

A total of 52 follicles were included in the three-dimensional analysis. Follicles were visualised across three orthogonal planes (XY, XZ, and YZ) (Fig. 3B and C). At each plane, follicles were classified as having only fpGCs (F) or a mixture of fpGCs and cuboidal GCs (C). Based on this analysis, follicles were classified as primordial (only fpGCs across all three planes, FFF) or transitional (cuboidal GCs observed in at least one plane). Transitional follicles were further classified as having GCs at one (FFC), two (FCC), or all three planes (CCC). For each follicle and oocyte, the diameter (largest and perpendicular diameter on the XY plane) and depth (diameter across the z-axis) were measured, in addition to fpGC and GC height at each plane.

### Presence of cuboidal granulosa cells

**Figure 4:**
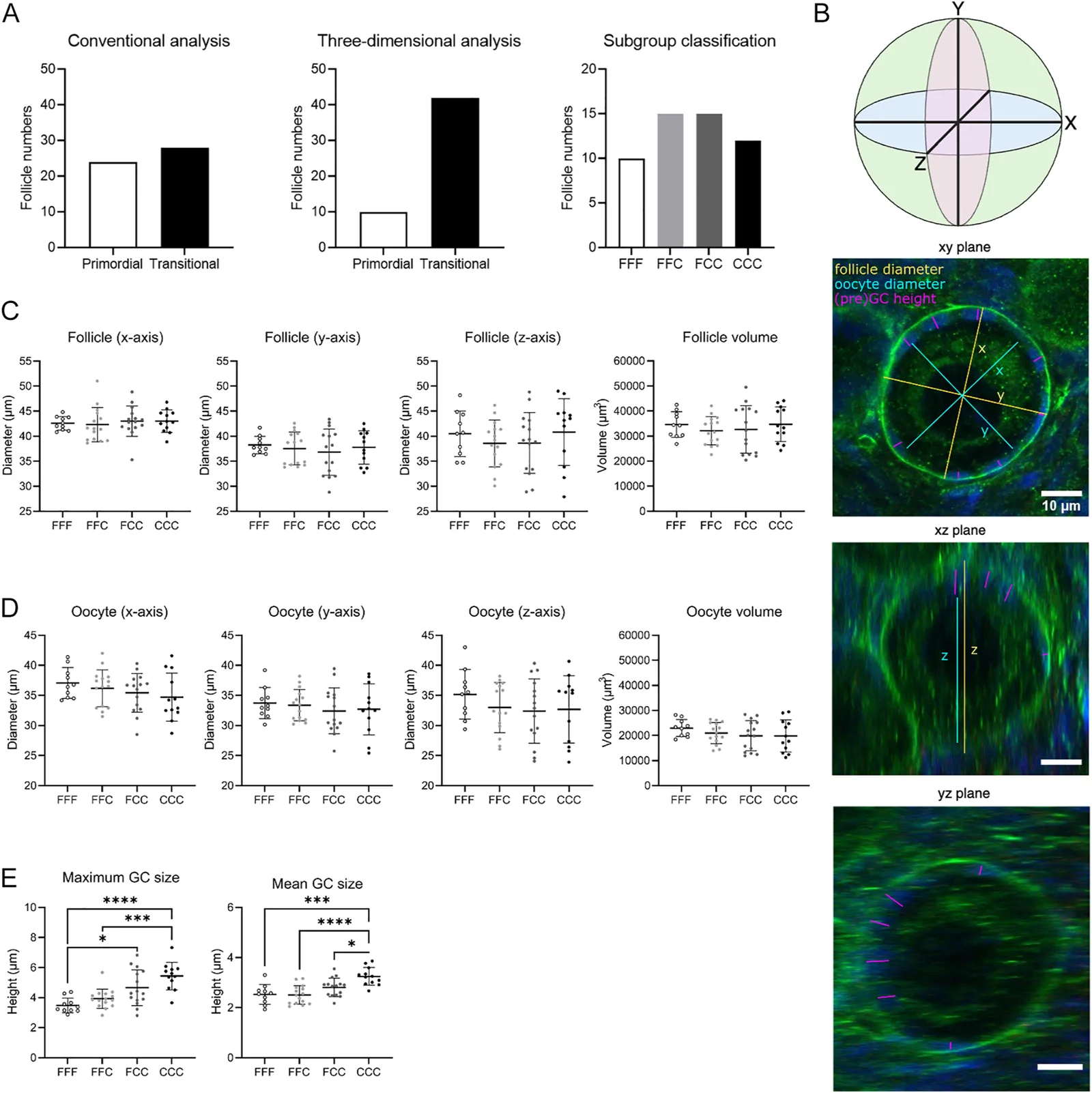
Three-dimensional analysis of primordial and transitional follicles. Fifty-two follicles were classified and analysed across three planes (XY, XZ, YZ). Follicles were further classified into subgroups based on whether only flattened pre-granulosa cells (fpGCs) were observed across all three planes (FFF, primordial) or a mixture of fpGCs and cuboidal granulosa cells (GCs) were observed at one (FFC, transitional), two (FCC, transitional), or all three planes (CCC, transitional), as described in the text. (A) Follicles were analysed and identified as primordial and transitional follicles based on the largest follicle cross-section across the XY plane (conventional analysis) or across three planes (three-dimensional analysis). (B) Diagram of an ellipsoid to represent a three-dimensional view of a follicle across three planes: X, Y, and Z. A representative image of a follicle (classified as FCC) is shown. For each follicle, the maximum follicle (yellow) and oocyte (blue) diameter was measured (defined as the x-axis), along with the corresponding perpendicular diameter (y-axis). The depth of the follicle was also measured across the z-axis. The height of (pre-)granulosa cells was measured (magenta lines) across all three planes (XY, XZ, and YZ). Scale bars represent 10 μm. (C) Follicle diameter (x- and y-axis; μm), follicle depth (z-axis; μm), and follicle volume (μm^3^). (D) Oocyte diameter (x- and y-axis; μm), oocyte depth (z-axis; μm), and oocyte volume (μm^3^). (E) Maximum and mean (pre-)GC height. *P < 0.05, ***P < 0.001, ****P < 0.0001. Created using BioRender.com.

When viewed only across the XY plane (as in conventional analysis), 24 follicles were classified as primordial and 28 as transitional (Fig. 4A). However, when viewed across all three planes, only ten follicles were found to be primordial and 42 were transitional; therefore, 14 follicles were misclassified. Using the conventional staging method, 58% (14 of 24) of follicles initially classified as primordial contained cuboidal GCs that could not be observed across the XY plane and were therefore actually transitional follicles. Out of the 42 confirmed transitional follicles identified with our three-dimensional method, only 12 follicles contained one or more cuboidal cells at all three planes.

### Follicle and oocyte diameter

Follicle and oocyte diameters were measured across three axes (X, Y, and Z) and volume calculated (Fig. 4B). There was no difference in follicle size (diameter across the X, Y, and Z axes) or volume between the four groups, and no difference in oocyte size or volume (Fig. 4C and D).

### Granulosa cell size

There was a significant difference in both mean and maximum fpGC size between the different stages of follicle development (Fig. 4E). The maximum fpGC size was significantly smaller for FFF (primordial) follicles (3.5 ± 0.5 μm) compared to transitional follicles (FCC and CCC, 4.6 ± 1.2 μm with *P* < 0.05 and 5.4 ± 0.9 μm with *P* < 0.0001, respectively). Within transitional follicles, there was also a difference in maximum fpGC cell height between FFC and CCC follicles (3.9 ± 0.6 μm vs 5.4 ± 0.9 μm with *P* < 0.001). Furthermore, there was a significant difference in mean fpGC size between the groups. The mean fpGC size of FFF follicles was significantly smaller than transitional follicles (CCC group, 2.5 ± 0.4 μm compared to 3.2 ± 0.4 μm with *P* < 0.001). Within transitional follicles, there was a significant difference in mean fpGC size between FFC and CCC (*P* < 0.0001) and FCC and CCC (*P* < 0.05) follicles (FFC: 2.5 ± 0.4 μm, FCC: 2.8 ± 0.4 μm, and CCC: 3.2 ± 0.4 μm).

## Discussion

The primary aim of this novel study was to develop a three-dimensional whole-mount immunolabelling and optical tissue clearing technique to comprehensively visualise human ovarian follicles for the purpose of improved classification at the primordial and transitional stages.

According to the results, our method enabled more accurate follicle classification, revealing that 58% of follicles initially classified as primordial using conventional two-dimensional (XY plane) analysis were reclassified as transitional when assessed with our three-dimensional (XY, XZ, and YZ planes) approach. This staggering discrepancy underscores the limitations of two-dimensional histology, which captures follicular features in a single plane and may miss key morphological features, such as the presence of cuboidal granulosa cells that define transitional follicles. GC size was determined by diameter measurement as opposed to volume to differentiate between fpGCs and cuboidal GCs. This measurement was selected as it applies in both conventional analysis and the three-dimensional technique provided here. In this study, primordial and transitional follicles did not differ in follicle nor oocyte diameter, yet there was a difference in fpGC and GC size. Thus, a comprehensive view of all follicular GCs across all three planes is required for accurate follicle classification and subgroup analysis.

This is the first study to describe three-dimensional analysis of human primordial and transitional follicles with optical tissue clearing. Previously, three-dimensional analysis of mouse and bovine follicle size has been reported using either serial histological sections (Van Wezel & Rodgers 1996) or tissue clearing (Feng *et al.* 2017), without measurements or detailed assessment of fpGCs. Methods to optically clear human ovarian tissue have been reported to identify follicle viability (Barbato *et al.* 2023) and assess protein expression (Biswas *et al.* 2021); however, follicle classification was not assessed. Previously described spatial analysis of growing human ovarian follicles was limited to the XY plane (Quan *et al.* 2020). The whole-mount immunolabelling and tissue clearing technique used in the present study enables the investigation of spatial effects across three dimensions using a combination of DAPI, DDX4, and laminin. Follicle health and incidence of atresia were not used as endpoints in this study; however, this methodology paves the way for future studies to investigate markers of follicle development.

The ability to distinguish between primordial and transitional follicles has important scientific and clinical implications. Biologically, this transition marks the earliest shift from dormancy (primordial stage) to activation, involving both significant transcriptomic and morphological changes (Maciel *et al.* 2004, McLaughlin & McIver 2009, Zhang *et al.* 2018, Rooda *et al.* 2024, Kubo *et al.* 2025). Follicle activation governs the rate of recruitment and depletion of the ovarian reserve, with accurate staging vital for investigating *in vitro* growth strategies for fertility preservation (Yang *et al.* 2020, Telfer & Andersen 2021). Clinically, assessing ovarian damage from chemotherapy depends on reliable classification of quiescent versus activated follicles, as current hypotheses suggest loss may occur either through premature activation (‘burnout’) (Erden & Oktay 2025) or direct apoptosis of dormant follicles (Titus *et al.* 2021). By preserving three-dimensional follicular architecture, our approach enables more precise and reproducible analysis of early-stage follicle development.

Human ovarian tissue fragments were successfully rendered transparent, with the tissue visualised up to ∼250 μm depth by imaging each sample from both sides. This limitation in z-depth is likely due to the dense nature of the human ovarian cortex (Richardson & Lichtman 2015). The Visikol® HISTO™ technique does not fully remove lipids from the tissue, a potential source of light scattering. Alternative clearing methods could improve this limitation. However, other tissue clearing methods can cause tissue shrinkage or swelling, which may affect follicle morphology (Richardson & Lichtman 2015). Furthermore, mechanical sectioning of paraffin-embedded tissue would no longer be required, mitigating variability of user ability. Data collection could be expedited as tissue sections would not have to be imaged and analysed individually, but in one instance using z-stacks. Visikol® HISTO™ has previously been used to generate three-dimensional renderings of immunolabelled non-human primate and human placental tissue (Merz *et al.* 2018, Sargent *et al.* 2019); other tissue clearing methods have been applied to investigate human ovaries (Biswas *et al.* 2021, Barbato *et al.* 2023). However, there are no reports of this technology being used to classify human ovarian follicles.

In the present study, most follicles analysed in tissue fragments cultured for 2 days were at the transitional stage. This is in line with reports from both our own group (Bjarkadottir *et al.* 2021, Walker *et al.* 2021) and others (Pampanini *et al.* 2019, Lopes *et al.* 2020, De Bruin *et al.* 2002). Follicular analysis was restricted to primordial and transitional follicles, but this method could more accurately classify other follicles, such as those at the primary, secondary, and pre-antral stages. Follicle stage distributions should not be extrapolated to uncultured (‘day 0’) tissue without direct comparison. Short-term culture (up to 8 days) of human ovarian cortex, and even the act of fragmenting cortical tissue, has been reported to promote activation of dormant primordial follicles, which would result in shifting the distribution between stages (Anderson *et al.* 2018, Kagami *et al.* 2018, McLaughlin *et al.* 2018, Bjarkadottir *et al.* 2021, Walker *et al.* 2021, Barbato *et al.* 2023). Accordingly, the ratio of primordial to transitional follicles observed here in cultured fragments is likely to differ from that of fresh (‘day 0’) tissue (Anderson *et al.* 2018, Bjarkadottir *et al.* 2021, Walker *et al.* 2021, Barbato *et al.* 2023). We also note that the supporting literature uses conventional histology, which may call into question the true recorded number of follicles at each stage (Bjarkadottir *et al.* 2021, Walker *et al.* 2021, Barbato *et al.* 2023). Further investigation with a larger number of patients analysing cultured and uncultured tissues would be required to confirm follicle proportions.

While we appreciate that histology is considered the gold standard for assessing ovarian follicle development, our study was specifically designed to evaluate the contribution of three-dimensional visualisation using confocal imaging. We did this by comparing two-dimensional and three-dimensional views within the same immunolabelled tissue block, thus preserving tissue architecture and avoiding artefacts introduced by the fixation, embedding, and sectioning processes. Utilising optical sectioning via confocal microscopy enabled a controlled assessment of dimensional influences on follicle classification. It is important to note that a direct comparison with standard histology of the same tissues and finding the same primordial follicles would be impossible; moreover, we do not consider this to be as accurate as comparison using the same method.

Shrinkage of tissue during preparation for analysis is a well-known occurrence. The standard histological workflow of formalin-fixed, paraffin-embedded processing can cause tissue shrinkage of up to 15%, and solvent-based dehydration has also been reported to cause shrinkage (Jali *et al.* 2015, Richardson & Lichtman 2015, Tran *et al.* 2015). Shrinkage due to clearing was not quantified in this study; however, shrinkage would not change the morphology of the primordial and transitional follicles and thus would not change the results of this study.

In this study, we focused on pre-pubertal ovarian cortical tissue due to its high density of early-stage follicles, particularly primordial and transitional stages, which are the primary targets of our classification method (Overland *et al.* 2023). While older tissue is indeed important for studying broader ovarian physiology, early-stage follicles are present across both pre- and post-pubertal tissues (Hansen *et al.* 2011, Overland *et al.* 2023). Paediatric and adolescent cortical tissues represent a growing proportion (approximately 15–40%) of cryopreserved samples, likely due to increased uptake of fertility preservation (Jadoul *et al.* 2017, Poirot *et al.* 2019, Emrich *et al.* 2025). Further studies including larger numbers of tissues and follicles from individuals with different histories and ages are required to validate the robustness of our protocol. For example, it will be important to assess potential age-related differences in tissue characteristics, such as antibody penetration or stromal density, which may affect protocol performance (Amargant *et al.* 2020).

In summary, the focus of this study was to demonstrate the potential of our novel protocol for identifying and quantifying primordial and transitional follicles in whole human tissue. Visualisation of follicular structures across all three dimensions is vital when distinguishing between primordial and transitional follicles. While the size of fpGCs differed between primordial and transitional follicles and within transitional follicle subgroups, there was no difference in follicle or oocyte size or volume. Histological analysis remains fundamental in studying ovarian tissue, yet recent advancements enable three-dimensional visualisation and analysis, offering significant potential for advancing ovarian research.

## Supplementary materials







## Declaration of interest

The authors declare that there is no conflict of interest that could be perceived as prejudicing the impartiality of the work reported.

## Funding

This project was supported by the Oxford Medical Research Council Doctoral Training Programme10.13039/501100000265 (Oxford MRC-DTP) grant (MR/N013468/1) awarded to BDB, and the Iceland Chamber of Commerce to BDB, the Icelandic Cancer Society to SAW and BDB. This project has also been made possible in part by grant number 2021-238038 from the Chan Zuckerberg Initiative DAF, an advised fund of the Silicon Valley Community Foundation10.13039/100014989.

## Author contribution statement

ASH was responsible for writing the original draft, writing review and editing, and data curation. BDB was responsible for writing the original draft, conceptualisation, formal analysis, investigation, methodology, and funding acquisition. NK was responsible for writing review and editing, and writing the original draft. JD and SL helped in sample acquisition. SAW was responsible for resources, supervision, conceptualisation, project administration, and funding acquisition.
