# Supplementary material for: Three-dimensional visualisation of human ovarian follicles using a whole-mount immunolabelling and optical tissue clearing method
Source: Reprod Fertil. 2025 Oct 31;6(4):e250096. doi: 10.1530/RAF-25-0096 (PMC12957915; doi:10.1530/RAF-25-0096)
Supplement: Supplementary file 1 [file supplementary_materials.pdf]

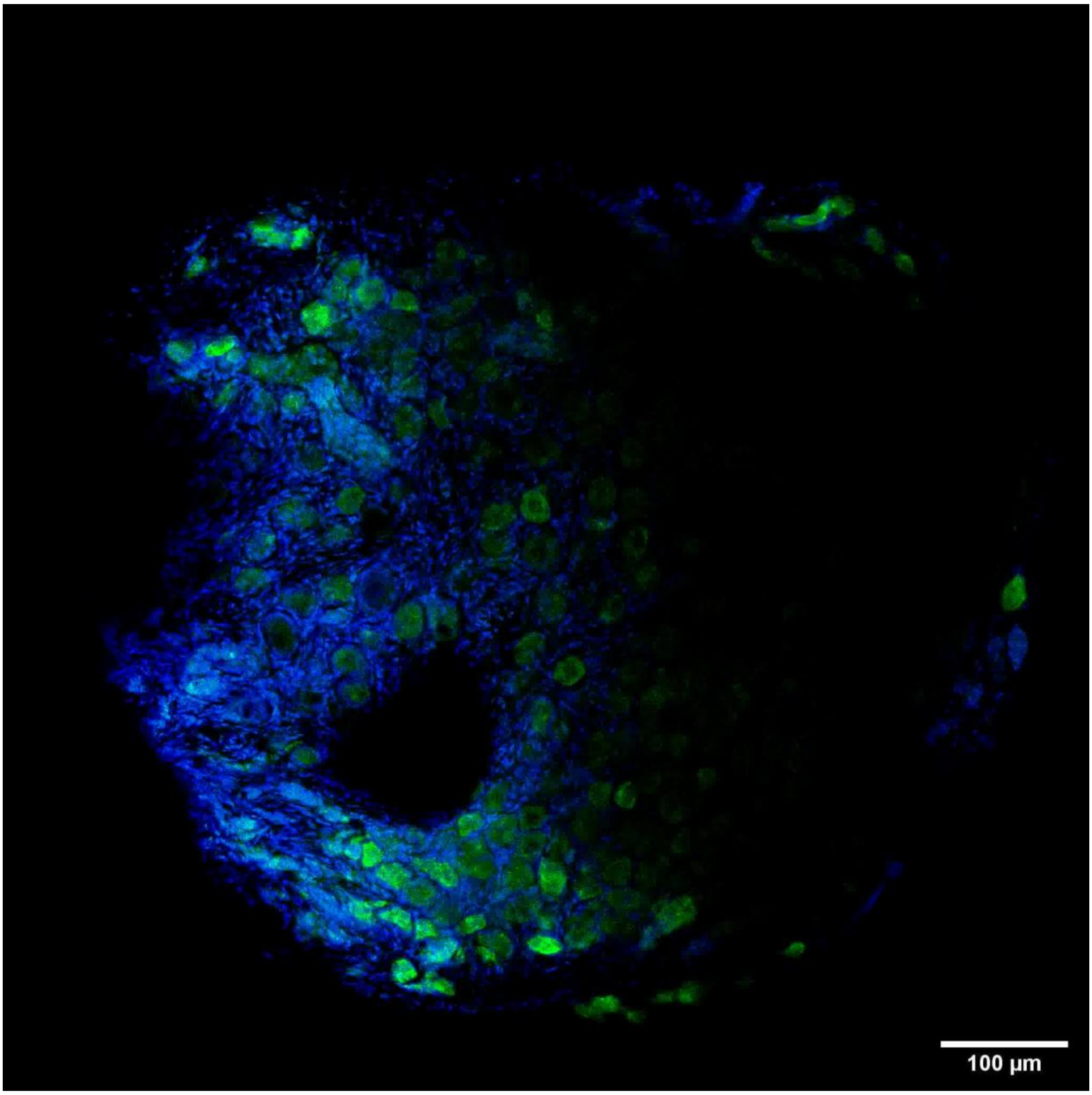

Video 1 STILL. Follicles present in optically cleared human ovarian tissue Z-stacks. Human ovarian tissue whole-mount immunolabelled with DEAD-Box Helicase 4 (DDX4) antibody for oocytes (green) as a marker for follicles and counterstained with DAPI (blue). Series of z-stack (optical sectioning; 3 frames per second) images at 10 Î¼m intervals with a 10x objective.

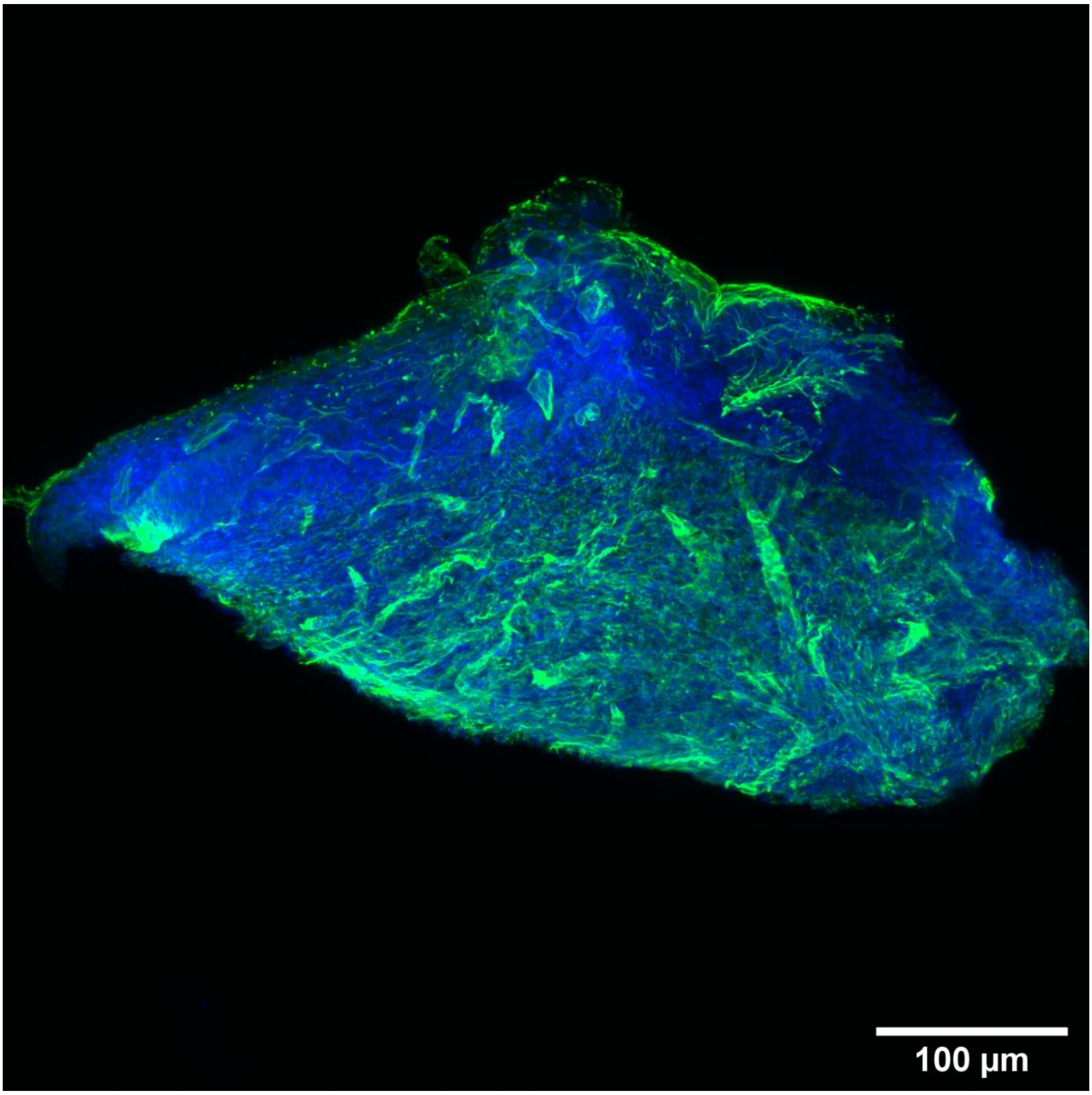

Video 2 STILL. Three-dimensional projection of optically cleared human ovarian tissue. Whole-mount immunolabelled with DEAD-BOX Helicase 4 (DDX4) and laminin in green for oocytes and basement membrane, respectively. Sample counterstained with DAPI (blue). Projection of z-stacks imaged at 10 Î¼m intervals with a 10x objective.
